# Supplementary material for: The Evolution and Characterization of the RNA Interference Pathways in Lophotrochozoa
Source: Genome Biol Evol. 2024 May 7;16(5):evae098. doi: 10.1093/gbe/evae098 (PMC11114477; doi:10.1093/gbe/evae098)
Supplement: evae098_Supplementary_Data [file evae098_supplementary_data.zip › Supplementary_materials.zip!/selected_miRNAs/NoGe_selected_miRNAs/mirdeep_pdf_files/NMRB01001254.1_Notospermus_geniculatus_strain_Ushimado_scaffold1254,_whole_genome_shotgun_sequence_26720.pdf]

Provisional ID	:	NMRB01001254.1_Notospermus_geniculatus_strain_Ushimado_scaffold1254,_whole_genome_shotgun_sequence_26720
Score total	:	88862.4
Score for star read(s)	:	3.9
Score for read counts	:	88854
Score for mfe	:	1.5
Score for randfold	:	0
Score for cons. seed	:	3
Total read count	:	174295
Mature read count	:	174095
Loop read count	:	0
Star read count	:	200

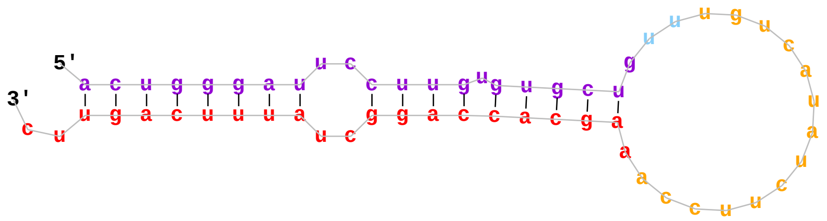

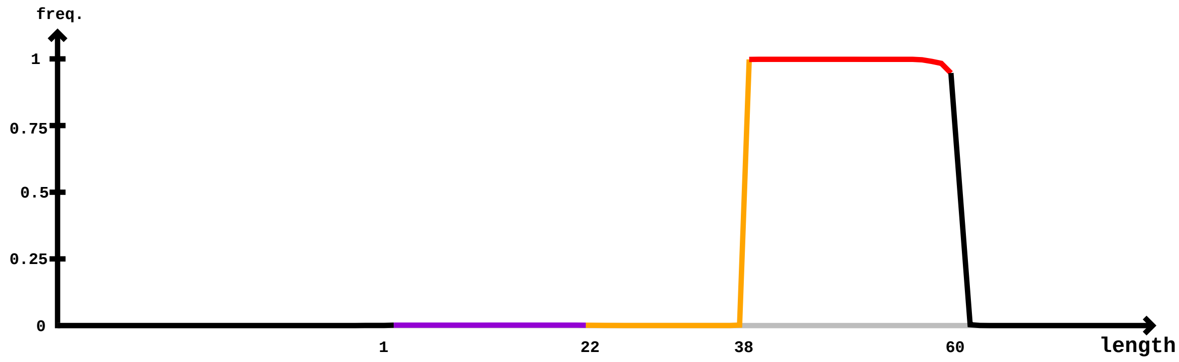


```
                                 Star                                   Mature
5'- agccagaacggagcugcugaaccuauccaguggacugggauuccuugugugcuguuugucauaucuuccaaagcaccaggcuauuucaguucucugguugggagaauuccac  -3'   obs
    agccagaacggagcugcugaaccuauccaguggacugggauuccuugugugcuguuugucauaucuuccaaagcaccaggcuauuucaguucucugguugggagaauuccac        exp
    .........(((....((...((((.((((.((((((((((..((((.(((((..................)))))))))..)))))))))).)))).))))))...)))..    reads    mm    sample
    ...........agcugcugaaccuauccagugg...............................................................................    1        0     Fm3
    ..............................uggacugggauuccuugugugcu...........................................................    30       0     Fm3
    ..............................uggacugggUuuccuugugugcu...........................................................    1        1     Fm3
    .................................acugggauuccuugugugcug..........................................................    98       0     Fm3
    .................................acugggauuccuuguguUcug..........................................................    2        1     Fm3
    .................................acugggauuccuugugAgcug..........................................................    1        1     Fm3
    .................................acugggauuccuugugCgcug..........................................................    1        1     Fm3
    .................................acugggauuccuugugugcugu.........................................................    9        0     Fm3
    .................................acugggauuccuugugugcuguu........................................................    9        0     Fm3
    .................................acugggauuccuugugugcuguuu.......................................................    1        0     Fm3
    .................................acugggauuccuugugugcuguuug......................................................    4        0     Fm3
    .....................................................................aaagcaccaggcuauuucag.......................    2        0     Fm3
    .....................................................................aaagcaccaggcuauuucaguuc....................    1        0     Fm3
    ......................................................................aagcaccaggcuauuucU........................    2        1     Fm3
    ......................................................................aagcaccaggcuauuuca........................    30       0     Fm3
    ......................................................................aagcacAaggcuauuucag.......................    1        1     Fm3
    ......................................................................aagcaccGggcuauuucag.......................    1        1     Fm3
    ......................................................................aagcaccaggcuauuucaA.......................    3        1     Fm3
    ......................................................................aagcaccaggcuauuuAag.......................    4        1     Fm3
    ......................................................................aagcaccaggcuauuucGg.......................    3        1     Fm3
    ......................................................................aagcaccaggcuauuucag.......................    678      0     Fm3
    ......................................................................aagcaccaggcuauuucUg.......................    1        1     Fm3
    ......................................................................aagcaccaggAuauuucag.......................    1        1     Fm3
    ......................................................................aagcaccaggcuauuuUag.......................    3        1     Fm3
    ......................................................................aagcaccaggcuauuucagu......................    141      0     Fm3
    ......................................................................aagcaccaggcuauuucagA......................    1        1     Fm3
    ......................................................................aagcaccaggcuauuucUgu......................    1        1     Fm3
    ......................................................................aagcaccaggcuauuucGgu......................    1        1     Fm3
    ......................................................................aagcaccaggcuaCuucagu......................    1        1     Fm3
    ......................................................................aagcaccaggcuauuucUguu.....................    2        1     Fm3
    ......................................................................aagcaccaggcuauuuGaguu.....................    2        1     Fm3
    ......................................................................aagcaccaggcuauuuAaguu.....................    1        1     Fm3
    ......................................................................aagcaccaggcuauuucaguA.....................    2        1     Fm3
```

Star Mature

```
agccagaacggagcugcugaaccuauccaguggacugggauuccuugugugcuguuugucauaucuuccaaagcaccaggcuauuucaguucucugguugggagaauuccac
.....................................................................aagcaccaggcuauuucaguu..................... 401 0 Fm3
.....................................................................aagcaccaggAuauuucaguu..................... 1 1 Fm3
.....................................................................aagcaccaggcuUuuucaguu..................... 1 1 Fm3
.....................................................................aagcaccaggcuauuucCguu..................... 1 1 Fm3
.....................................................................aagcaccaggcuauuuUaguu..................... 4 1 Fm3
.....................................................................aaUcaccaggcuauuucaguu..................... 1 1 Fm3
.....................................................................aagcaccaggcuauuucGguu..................... 1 1 Fm3
.....................................................................aagcaccaggUuauuucaguuc.................... 7 1 Fm3
.....................................................................aagcacUaggcuauuucaguuc.................... 1 1 Fm3
.....................................................................aagcaccaggcuaAuucaguuc.................... 1 1 Fm3
.....................................................................aagcUccaggcuauuucaguuc.................... 1 1 Fm3
.....................................................................Gagcaccaggcuauuucaguuc.................... 1 1 Fm3
.....................................................................Nagcaccaggcuauuucaguuc.................... 1 1 Fm3
.....................................................................aagcaccaggcuauuucUguuc.................... 6 1 Fm3
.....................................................................aagcaccaggcuUuuucaguuc.................... 1 1 Fm3
.....................................................................aagcaccaggcuauuucaguuU.................... 20 1 Fm3
.....................................................................aagGaccaggcuauuucaguuc.................... 1 1 Fm3
.....................................................................aagcaccaggcuauuuGaguuc.................... 3 1 Fm3
.....................................................................aagcaccaggcuauuuUaguuc.................... 6 1 Fm3
.....................................................................aagcaccaUgcuauuucaguuc.................... 1 1 Fm3
.....................................................................aagcaccaggcuauuucaguAc.................... 2 1 Fm3
.....................................................................aagcaccaggcuauuucaguuA.................... 7 1 Fm3
.....................................................................aagcaUcaggcuauuucaguuc.................... 2 1 Fm3
.....................................................................aaUcaccaggcuauuucaguuc.................... 2 1 Fm3
.....................................................................Uagcaccaggcuauuucaguuc.................... 2 1 Fm3
.....................................................................aagcaccaggAuauuucaguuc.................... 5 1 Fm3
.....................................................................aagcaccaggGuauuucaguuc.................... 5 1 Fm3
.....................................................................aagcaccaggcuauCucaguuc.................... 3 1 Fm3
.....................................................................aGgcaccaggcuauuucaguuc.................... 1 1 Fm3
.....................................................................aagcaccaggcuauuucaguuc.................... 2549 0 Fm3
.....................................................................aagcaccaggcuauuucCguuc.................... 4 1 Fm3
.....................................................................aagcaccaggcuauuCcaguuc.................... 1 1 Fm3
.....................................................................aagcaccaggcuauuucaguuG.................... 1 1 Fm3
.....................................................................aagcaccaggcuauuuAaguuc.................... 8 1 Fm3
.....................................................................aagcaccaggcuauuucGguuc.................... 6 1 Fm3
.....................................................................aagcaccaggcuauuucaguucC................... 7 1 Fm3
.....................................................................aagcaccaggcuauuucaguucu................... 167 0 Fm3
.....................................................................aaAcaccaggcuauuucaguucu................... 1 1 Fm3
.....................................................................aagcaccaggcuauCucaguucu................... 1 1 Fm3
.....................................................................aagcaccaggcuauuucaguuUu................... 1 1 Fm3
.....................................................................aagcaccaggcuauuucaguucA................... 119 1 Fm3
.....................................................................aagcaccaggcuauuucaguucuU.................. 1 1 Fm3
.....................................................................aagcaccaggcuauuucaguucuA.................. 1 1 Fm3
......................................................................agcaccaggcuauuucaguu..................... 1 0 Fm3
......................................................................agcaccaggcuauuucaguuc.................... 8 0 Fm3
......................................................................agcaccaggcuauuucaguucu................... 1 0 Fm3

.............................uggacugggauuccuugugugcu........................................................... 7 0 Mm3
.............................uggacugggauuccuugugugcuU.......................................................... 1 1 Mm3
................................acugggauuccuugugugcug.......................................................... 3 0 Mm3
................................acugggauuccuugugugcugu......................................................... 2 0 Mm3
................................acugggauuccuugugugcuguu........................................................ 8 0 Mm3
................................acugggauuccuugugugcuguuu....................................................... 8 0 Mm3
................................acugggauuccuugugugcuguuug...................................................... 3 0 Mm3
................................acugggauuccuugugugcuguuuguc.................................................... 2 0 Mm3
............................................ugugugcuguuugucauaucuucca.......................................... 1 0 Mm3
.......................................................ugucauaucuuccaaagcaccaggcu.............................. 1 0 Mm3
.........................................................ucauaucuuccaaagcaccaggc............................... 2 0 Mm3
.........................................................ucauaucuuccaaagcaccaggcu.............................. 4 0 Mm3
.........................................................ucauaucuuccaaagcaccaggcuauuucaguuc.................... 1 0 Mm3
.........................................................ucauaucuuccaaagcaccaggcuauuucaguucu................... 1 0 Mm3
....................................................................aaagcaccaggcuauuucaguu..................... 2 0 Mm3
....................................................................aaagcaccaggcuauuucaguuc.................... 4 0 Mm3
....................................................................Caagcaccaggcuauuucaguuc.................... 2 1 Mm3
.....................................................................aagcaccaggcuauuuca........................ 22 0 Mm3
.....................................................................aagcaccaggcuauuucaA....................... 1 1 Mm3
.....................................................................aagcaccaggcuauuucag....................... 16 0 Mm3
.....................................................................aagcaccaggcuauuucagu...................... 82 0 Mm3
.....................................................................aagcaccaggUuauuucagu...................... 1 1 Mm3
.....................................................................aagcaccaggcuauuuUaguu..................... 1 1 Mm3
```

Star **Mature**

```
agccagaacggagcugcugaaccuauccaguggacugggauuccuugugugcuguuugucauaucuuccaaagcaccaggcuauuucaguucucugguugggagaauuccac
......................................................................aagcacUaggcuauuucaguu.....................   1      1    Mm3
......................................................................aagcaccaggcuauuuAaguu.....................   1      1    Mm3
......................................................................aagcGccaggcuauuucaguu.....................   1      1    Mm3
......................................................................Uagcaccaggcuauuucaguu.....................   1      1    Mm3
......................................................................aagcaccaggcuauuucaguu.....................   354    0    Mm3
......................................................................aagcaccaggcuauuucaguA.....................   1      1    Mm3
......................................................................aagcaccaggcuauuucUguu.....................   4      1    Mm3
......................................................................aagcaccaggcuGuuucaguuc....................   1      1    Mm3
......................................................................Gagcaccaggcuauuucaguuc....................   1      1    Mm3
......................................................................aagcaccaggUuauuucaguuc....................   4      1    Mm3
......................................................................aagcaccaggcuauuCcaguuc....................   1      1    Mm3
......................................................................aagcaccaggcuauuucGguuc....................   20     1    Mm3
......................................................................aCgcaccaggcuauuucaguuc....................   2      1    Mm3
......................................................................aagcaccaggcuauuucaguuU....................   36     1    Mm3
......................................................................aagcaAcaggcuauuucaguuc....................   1      1    Mm3
......................................................................aagcaccaggcuauuuUaguuc....................   20     1    Mm3
......................................................................aaAcaccaggcuauuucaguuc....................   3      1    Mm3
......................................................................aagcaccCggcuauuucaguuc....................   1      1    Mm3
......................................................................aagcaccaUgcuauuucaguuc....................   2      1    Mm3
......................................................................Nagcaccaggcuauuucaguuc....................   6      1    Mm3
......................................................................aagcaccagAcuauuucaguuc....................   2      1    Mm3
......................................................................aagcaccGggcuauuucaguuc....................   1      1    Mm3
......................................................................aagcaccaggcuauuucUguuc....................   14     1    Mm3
......................................................................aagcaccaggAuauuucaguuc....................   6      1    Mm3
......................................................................aagcaccaggcuauuucaguCc....................   2      1    Mm3
......................................................................aagcaccUggcuauuucaguuc....................   2      1    Mm3
......................................................................aagcaccaggcuauuucaguuc....................   5522   0    Mm3
......................................................................aagcaccaggcuauuuGaguuc....................   4      1    Mm3
......................................................................aagcaUcaggcuauuucaguuc....................   2      1    Mm3
......................................................................aaUcaccaggcuauuucaguuc....................   2      1    Mm3
......................................................................aagcaccaggcuauuucaguuA....................   10     1    Mm3
......................................................................aagcaccaggGuauuucaguuc....................   8      1    Mm3
......................................................................aagcaccaggcuauuucaUuuc....................   2      1    Mm3
......................................................................aGgcaccaggcuauuucaguuc....................   1      1    Mm3
......................................................................aagcaNcaggcuauuucaguuc....................   1      1    Mm3
......................................................................Uagcaccaggcuauuucaguuc....................   3      1    Mm3
......................................................................aagcaccaggcuauuucCguuc....................   17     1    Mm3
......................................................................aagcaccaggcuUuuucaguuc....................   2      1    Mm3
......................................................................aagcaccaggcuauuuAaguuc....................   25     1    Mm3
......................................................................aagcaccaggcuauuucagGuc....................   1      1    Mm3
......................................................................aagcaccaggcuaAuucaguuc....................   1      1    Mm3
......................................................................aagcacUaggcuauuucaguuc....................   4      1    Mm3
......................................................................aagcaccaggcuauuucaguAc....................   2      1    Mm3
......................................................................aagcaccaggcuauuucaguucG...................   1      1    Mm3
......................................................................aagcaccaggGuauuucaguucu...................   1      1    Mm3
......................................................................aagcaccaggcuauuucCguucu...................   1      1    Mm3
......................................................................aagcacUaggcuauuucaguucu...................   1      1    Mm3
......................................................................aagcaccaggcuauuucUguucu...................   1      1    Mm3
......................................................................aagUaccaggcuauuucaguucu...................   1      1    Mm3
......................................................................aagcaccaggcuauuuAaguucu...................   1      1    Mm3
......................................................................aagcaccaggcuauuucaguuAu...................   1      1    Mm3
......................................................................aagcaccaggcuauuucaguucC...................   5      1    Mm3
......................................................................aaCcaccaggcuauuucaguucu...................   1      1    Mm3
......................................................................aagcaccaggcuauuuUaguucu...................   1      1    Mm3
......................................................................aagcaccaggcuauuucaguucu...................   444    0    Mm3
......................................................................aagcaNcaggcuauuucaguucu...................   1      1    Mm3
......................................................................aagcaccaggcuauuucGguucu...................   2      1    Mm3
......................................................................aagcaccaggcuauuucaguuUu...................   2      1    Mm3
......................................................................aagcaccaggcuauuucaguucA...................   237    1    Mm3
......................................................................aagcaccaggcuauuuGaguucu...................   1      1    Mm3
......................................................................aagcaccaggcuauuucaguucuA..................   2      1    Mm3
......................................................................aagcaccaggcuauuucaguucuU..................   23     1    Mm3
......................................................................aagcaccaggcuauuucaguucuUu.................   5      1    Mm3
.......................................................................agcaccaggcuauuucaguu.....................   1      0    Mm3
.......................................................................agcaccaggcuauuucaguuc....................   5      0    Mm3
.......................................................................agcaccaggcuauuucaguucu...................   1      0    Mm3
........................................................................gcaccaggcuauuucaguuc....................   1      0    Mm3

..............................uggacugggauuccuugugugcu...........................................................   1      0    Fm1
.................................acugggauuccuugugugcug..........................................................   3      0    Fm1
```

Star **Mature**

```
agccagaacggagcugcugaaccuauccaguggacugggauuccuugugugcuguuugucauaucuuccaaagcaccaggcuauuucaguucucugguugggagaauuccac
.................................acugggauuccuugugugcGg..........................................................   1     1   Fm1
.................................acugggauuccuugugugcuguuu.......................................................   3     0   Fm1
...........................................cuugugugcuguuugucauaucuucca..........................................   4     0   Fm1
..........................................................ucauaucuuccaaagcaccaggcu..............................   1     0   Fm1
.....................................................................aaagcaccaggcuauuuAagu......................   1     1   Fm1
.....................................................................aaagcaccaggcuauuucagu......................   1     0   Fm1
.....................................................................Caagcaccaggcuauuucaguu.....................   4     1   Fm1
.....................................................................aaagcaccaggcuauuucaguG.....................   1     1   Fm1
.....................................................................aaagcacGaggcuauuucaguu.....................   1     1   Fm1
.....................................................................aaagcaccaggcuauuucaguu.....................   19    0   Fm1
.....................................................................Caagcaccaggcuauuucaguuc....................   18    1   Fm1
.....................................................................aaagcaccaggcuauuuAaguuc....................   1     1   Fm1
.....................................................................aaagcaccaggcuauuucaguuc....................   67    0   Fm1
.....................................................................Caagcaccaggcuauuucaguucu...................   5     1   Fm1
......................................................................aagcaccaggcuauuuAa........................   2     1   Fm1
......................................................................aagcaccaggcuauuuca........................   104   0   Fm1
......................................................................aGgcaccaggcuauuucag.......................   1     1   Fm1
......................................................................aagcaccaggcuauuuUag.......................   1     1   Fm1
......................................................................aagcaccaggcuauuucaU.......................   2     1   Fm1
......................................................................aagcaccaggcuauuucUg.......................   1     1   Fm1
......................................................................aagcaccaggcuauuucag.......................   137   0   Fm1
......................................................................aagcaccaggcuauuucaA.......................   1     1   Fm1
......................................................................aagcaccaggGuauuucagu......................   1     1   Fm1
......................................................................aagcaccaggcuauuucCgu......................   1     1   Fm1
......................................................................aagcaccaggcuauuucaUu......................   6     1   Fm1
......................................................................aagcaccaggcuauuucagA......................   6     1   Fm1
......................................................................Uagcaccaggcuauuucagu......................   1     1   Fm1
......................................................................aagcaccaggcuauuucagu......................   503   0   Fm1
......................................................................aagcaccaggcuauuucagC......................   1     1   Fm1
......................................................................aagcaccaggcuauuucGgu......................   2     1   Fm1
......................................................................aagcaccaggcuauuuAagu......................   1     1   Fm1
......................................................................aagcaccaggcuauuuUagu......................   2     1   Fm1
......................................................................aagcaccaggcuauuucUgu......................   1     1   Fm1
......................................................................aUgcaccaggcuauuucagu......................   1     1   Fm1
......................................................................aagcaccaggcuauuuGagu......................   1     1   Fm1
......................................................................Nagcaccaggcuauuucagu......................   1     1   Fm1
......................................................................aagcaccaggcuauuucaguA.....................   17    1   Fm1
......................................................................aagcaccaggcuauuuAaguu.....................   10    1   Fm1
......................................................................aagcGccaggcuauuucaguu.....................   2     1   Fm1
......................................................................aaCcaccaggcuauuucaguu.....................   2     1   Fm1
......................................................................aagcaccaggcuauuucaguG.....................   3     1   Fm1
......................................................................aagcacGaggcuauuucaguu.....................   1     1   Fm1
......................................................................aagcaccaggcuauuuUaguu.....................   6     1   Fm1
......................................................................aNgcaccaggcuauuucaguu.....................   1     1   Fm1
......................................................................aagcaccaggcuauuucagCu.....................   1     1   Fm1
......................................................................aaUcaccaggcuauuucaguu.....................   1     1   Fm1
......................................................................aagcaccaggcuauuucUguu.....................   4     1   Fm1
......................................................................aagcaccaggcuauuucCguu.....................   10    1   Fm1
......................................................................aagcaccaggcAauuucaguu.....................   1     1   Fm1
......................................................................aagcaccaAgcuauuucaguu.....................   1     1   Fm1
......................................................................aagcaccaggcuauuucaguu.....................   2501  0   Fm1
......................................................................aagcaccaggcuauuucGguu.....................   9     1   Fm1
......................................................................aagcaccaggUuauuucaguu.....................   5     1   Fm1
......................................................................aagcaccaggAuauuucaguu.....................   7     1   Fm1
......................................................................aagUaccaggcuauuucaguu.....................   1     1   Fm1
......................................................................aagcaccaggcuauuuGaguu.....................   5     1   Fm1
......................................................................aagcaccaggcuauuAcaguu.....................   1     1   Fm1
......................................................................aagcaccGggcuauuucaguu.....................   1     1   Fm1
......................................................................aagcaccaggcuauuCcaguu.....................   2     1   Fm1
......................................................................aagcaccaggcuaAuucaguu.....................   1     1   Fm1
......................................................................aagcUccaggcuauuucaguu.....................   1     1   Fm1
......................................................................aagcacUaggcuauuucaguu.....................   1     1   Fm1
......................................................................aagcaccaggcuauuucaguC.....................   1     1   Fm1
......................................................................aagcaccaggcCauuucaguu.....................   1     1   Fm1
......................................................................aGgcaccaggcuauuucaguu.....................   1     1   Fm1
......................................................................aagcaccaggGuauuucaguu.....................   2     1   Fm1
......................................................................aagcaccaggcuauuuGaguuc....................   75    1   Fm1
......................................................................aagcaccaggcuauAucaguuc....................   5     1   Fm1
......................................................................aagcaccaCgcuauuucaguuc....................   3     1   Fm1
......................................................................aagcacNaggcuauuucaguuc....................   5     1   Fm1
```

Star **Mature**

agccagaacggagcugcugaaccuauccaguggacugggauuccuugugugcuguuugucauaucuuccaaagcaccaggcuauuucaguucucugguugggagaauuccac			
....aagcaccUggcuauuucaguuc....................	18	1	Fm1
....aGgcaccaggcuauuucaguuc....................	15	1	Fm1
....aagcaccCggcuauuucaguuc....................	5	1	Fm1
....aagcaccagAcuauuucaguuc....................	7	1	Fm1
....aagcaccagUcuauuucaguuc....................	10	1	Fm1
....aagcaccaggcuauuCcaguuc....................	12	1	Fm1
....aagGaccaggcuauuucaguuc....................	1	1	Fm1
....aUgcaccaggcuauuucaguuc....................	5	1	Fm1
....aagcaNcaggcuauuucaguuc....................	11	1	Fm1
....aagAaccaggcuauuucaguuc....................	5	1	Fm1
....aagcaccaggcuauuucaguAc....................	2	1	Fm1
....aagcaccaggAuauuucaguuc....................	125	1	Fm1
....aagcaccaggcuauuucaguuG....................	6	1	Fm1
....aagcaccaggcGauuucaguuc....................	2	1	Fm1
....aagcaccaggcuCuucaguuc....................	3	1	Fm1
....Cagcaccaggcuauuucaguuc....................	3	1	Fm1
....aagcacGaggcuauuucaguuc....................	6	1	Fm1
....aagUaccaggcuauuucaguuc....................	2	1	Fm1
....aagcaccaAgcuauuucaguuc....................	4	1	Fm1
....aagcaccaggcuauuucaguuc....................	59669	0	Fm1
....aagcacAaggcuauuucaguuc....................	4	1	Fm1
....aagcaccaggcuauuucUguuc....................	184	1	Fm1
....aagcaccaggcuauuucaUuuc....................	9	1	Fm1
....aagcaccaggGuauuucaguuc....................	69	1	Fm1
....aagcacUaggcuauuucaguuc....................	22	1	Fm1
....aagcaUcaggcuauuucaguuc....................	23	1	Fm1
....aaCcaccaggcuauuucaguuc....................	11	1	Fm1
....aagcaccaggcuaCuucaguuc....................	10	1	Fm1
....aagcaccaggcuUuuucaguuc....................	4	1	Fm1
....aaUcaccaggcuauuucaguuc....................	12	1	Fm1
....Gagcaccaggcuauuucaguuc....................	13	1	Fm1
....aagcaccaggcuauCucaguuc....................	8	1	Fm1
....aagcaccaggcuauuucagCuc....................	5	1	Fm1
....aagcaccagCcuauuucaguuc....................	3	1	Fm1
....aagcaccaggcuaAuucaguuc....................	7	1	Fm1
....aagcCccaggcuauuucaguuc....................	5	1	Fm1
....aaAcaccaggcuauuucaguuc....................	7	1	Fm1
....aagcUccaggcuauuucaguuc....................	3	1	Fm1
....aagcaccaggcuauuAcaguuc....................	5	1	Fm1
....aagcaAcaggcuauuucaguuc....................	9	1	Fm1
....aagcaccaggcuauGucaguuc....................	3	1	Fm1
....aaNcaccaggcuauuucaguuc....................	1	1	Fm1
....aagcaccaggcuauuucaAuuc....................	3	1	Fm1
....aagcaccaggcuauuucaguCc....................	11	1	Fm1
....aagcaccaggcCauuucaguuc....................	6	1	Fm1
....aCgcaccaggcuauuucaguuc....................	2	1	Fm1
....aagcaccaggcuauuucaguuA....................	46	1	Fm1
....aagcaccaggcuaGuucaguuc....................	6	1	Fm1
....Nagcaccaggcuauuucaguuc....................	26	1	Fm1
....Uagcaccaggcuauuucaguuc....................	34	1	Fm1
....aagcaccaggcuauuucaCuuc....................	4	1	Fm1
....aagcaccaggcuGuucaguuc....................	2	1	Fm1
....aagcaccaggcuauuucCguuc....................	174	1	Fm1
....aagcaccaggcuauuucagAuc....................	4	1	Fm1
....aagcaccaUgcuauuucaguuc....................	11	1	Fm1
....aagcGccaggcuauuucaguuc....................	8	1	Fm1
....aagcaccaggUuauuucaguuc....................	54	1	Fm1
....aagcaccGggcuauuucaguuc....................	14	1	Fm1
....aagcaccaggcuauuucGguuc....................	172	1	Fm1
....aagcaGcaggcuauuucaguuc....................	3	1	Fm1
....aagcaccaggcuauuucagGuc....................	3	1	Fm1
....aagcaccaggcuauuUaguuc....................	137	1	Fm1
....aagcaccaggcuauuuAaguuc....................	224	1	Fm1
....aagcaccaggcAauuucaguuc....................	1	1	Fm1
....aagcaccaggcuauuucaguU....................	415	1	Fm1
....aagcaccaggcuauuucaAuucu...................	1	1	Fm1
....aagcaccaUgcuauuucaguucu...................	1	1	Fm1
....aagcaccaggcuauuucaguucC...................	173	1	Fm1
....aagcaccaggcuauuucaguucG...................	19	1	Fm1
....aagGaccaggcuauuucaguucu...................	3	1	Fm1

Star Mature

agccagaacggagcugcugaaccuauccaguggacugggauuccuugugugcuguuugucauaucuuccaaagcaccaggcuauuucaguucucugguugggagaauuccac			
....aagcaccaggcuauuucaguCcu...................	2	1	Fm1
....aagcaccaggcuauuucaguuUu...................	49	1	Fm1
....aaAcaccaggcuauuucaguucu...................	1	1	Fm1
....aagcaccaggcuauuucagCucu...................	4	1	Fm1
....aagcaccaggcuauuucaguuGu...................	6	1	Fm1
....aagcaccaggcuauuuAaguucu...................	17	1	Fm1
....aagcaccaggcCauuucaguucu...................	2	1	Fm1
....aagcaccaggcuauuucGguucu...................	11	1	Fm1
....aagcaccaggGuauuucaguucu...................	4	1	Fm1
....aagcaccaggcuauGucaguucu...................	1	1	Fm1
....aagcaccaggcuauuuGaguucu...................	2	1	Fm1
....aagcaccaggcuauuucCguucu...................	16	1	Fm1
....aagcaccaggUuauuucaguucu...................	9	1	Fm1
....aagcaccaggAuauuucaguucu...................	10	1	Fm1
....aagcaccUggcuauuucaguucu...................	1	1	Fm1
....aagcaccaggcuauuucUguucu...................	13	1	Fm1
....aagcaccagUcuauuucaguucu...................	1	1	Fm1
....aagcaccaggcuauuucaguucA...................	1764	1	Fm1
....aagcaccaggcuaGuucaguucu...................	1	1	Fm1
....Uagcaccaggcuauuucaguucu...................	1	1	Fm1
....aagcaUcaggcuauuucaguucu...................	2	1	Fm1
....aagcaccaggcuauuuUaguucu...................	9	1	Fm1
....aagcaccaggcuauuucaUuucu...................	1	1	Fm1
....aagcaccaggcuauuucaguucu...................	4584	0	Fm1
....Gagcaccaggcuauuucaguucu...................	2	1	Fm1
....aaUcaccaggcuauuucaguucu...................	2	1	Fm1
....aagcaccaggcuauuucaguuAu...................	12	1	Fm1
....aagcUccaggcuauuucaguucu...................	1	1	Fm1
....Nagcaccaggcuauuucaguucu...................	3	1	Fm1
....aagcacAaggcuauuucaguucu...................	1	1	Fm1
....aCgcaccaggcuauuucaguucu...................	1	1	Fm1
....aagcaccaggcuauuucagAucu...................	2	1	Fm1
....aagAaccaggcuauuucaguucu...................	1	1	Fm1
....aagcaccaggcuauuucaguucuc..................	1	0	Fm1
....aagcaccaggcuauuucaguucuA..................	18	1	Fm1
....aagcaccaggcuauuucaguucAc..................	2	1	Fm1
....aagcaccaggcuauuucaguucuU..................	239	1	Fm1
....aagcaccaggcuauuucaguucuUu.................	24	1	Fm1
....agcaccaggcuauuucaguuc....................	20	0	Fm1
....agcaccaggcuauuucagAuc....................	1	1	Fm1
....agcaccaggcuauuucaguucu...................	1	0	Fm1
....caccaggcuauuucaguucucu.................	3	0	Fm1
....aaagcaccaggcuauuucaguu.....................	7	0	Fm2
....aaagcaccaggcuauuuGaguu.....................	1	1	Fm2
....aaagcaccaggcuauuucaguuc....................	33	0	Fm2
....Caagcaccaggcuauuucaguuc....................	7	1	Fm2
....aagcaccaggcuauuuca........................	18	0	Fm2
....aagcaccaggcuauuucag.......................	33	0	Fm2
....aagcaccaggcuauuuAag.......................	1	1	Fm2
....aagcaccaggcuauuuGag.......................	1	1	Fm2
....aagcaccaggGuauuucag.......................	1	1	Fm2
....aagcaccaggcuauuucUgu......................	1	1	Fm2
....aagcaccaggcuauuuAagu......................	1	1	Fm2
....aagcaccagUcuauuucagu......................	1	1	Fm2
....Nagcaccaggcuauuucagu......................	1	1	Fm2
....aagcaccaggcuauuucGgu......................	1	1	Fm2
....aagcaccaggcuauuucagu......................	155	0	Fm2
....aagcaccaggcuauuucCgu......................	1	1	Fm2
....aagcaccagCcuauuucaguu.....................	1	1	Fm2
....aagcaccaggcuauuucGguu.....................	1	1	Fm2
....aagcaccaggGuauuucaguu.....................	1	1	Fm2
....Uagcaccaggcuauuucaguu.....................	1	1	Fm2
....aagcaccaggcuauuuAaguu.....................	3	1	Fm2
....aagcaccaggcuauuucaguu.....................	680	0	Fm2
....aagcaccaggcuauuucUguu.....................	3	1	Fm2
....aagcaccaggcuauuuGaguu.....................	1	1	Fm2
....aagcaccaggAuauuucaguu.....................	1	1	Fm2
....aagcaccaggcuauuuUaguu.....................	3	1	Fm2
....aGgcaccaggcuauuucaguu.....................	1	1	Fm2

Star Mature

```
agccagaacggagcugcugaaccuauccaguggacugggauuccuugugugcuguuugucauaucuuccaaagcaccaggcuauuucaguucucugguugggagaauuccac
......................................................................aagcaccaggcuauuucCguu.....................  1      1  Fm2
......................................................................aagcaccaggcuauuuGaguuc....................  29     1  Fm2
......................................................................aagcaccaggcuauuCcaguuc....................  7      1  Fm2
......................................................................aaAcaccaggcuauuucaguuc....................  8      1  Fm2
......................................................................aagcaccaggcuaAuucaguuc....................  9      1  Fm2
......................................................................aCgcaccaggcuauuucaguuc....................  5      1  Fm2
......................................................................aagcaccaggcuauuAcaguuc....................  3      1  Fm2
......................................................................aagcaccagCcuauuucaguuc....................  2      1  Fm2
......................................................................aagcUccaggcuauuucaguuc....................  5      1  Fm2
......................................................................aagcGccaggcuauuucaguuc....................  6      1  Fm2
......................................................................aaNcaccaggcuauuucaguuc....................  1      1  Fm2
......................................................................Cagcaccaggcuauuucaguuc....................  2      1  Fm2
......................................................................aagcCccaggcuauuucaguuc....................  3      1  Fm2
......................................................................aagcaAcaggcuauuucaguuc....................  1      1  Fm2
......................................................................aagcaccaggcuCuuucaguuc....................  1      1  Fm2
......................................................................Uagcaccaggcuauuucaguuc....................  11     1  Fm2
......................................................................aagcaccaggcAauuucaguuc....................  2      1  Fm2
......................................................................aagcaccaggAuauuucaguuc....................  44     1  Fm2
......................................................................aagcaccaUgcuauuucaguuc....................  10     1  Fm2
......................................................................aagcaccaggcuauuucGguuc....................  94     1  Fm2
......................................................................aagcaccaggcCauuucaguuc....................  2      1  Fm2
......................................................................aagcaGcaggcuauuucaguuc....................  1      1  Fm2
......................................................................aagcaccUggcuauuucaguuc....................  5      1  Fm2
......................................................................aagcaccaggcuauuuUaguuc....................  58     1  Fm2
......................................................................aagGaccaggcuauuucaguuc....................  1      1  Fm2
......................................................................aagcaccaAgcuauuucaguuc....................  1      1  Fm2
......................................................................aagcaccaggcuaGuucaguuc....................  2      1  Fm2
......................................................................aagcacNaggcuauuucaguuc....................  2      1  Fm2
......................................................................aagcaccGggcuauuucaguuc....................  3      1  Fm2
......................................................................aagcaccaggcuGuuucaguuc....................  3      1  Fm2
......................................................................aagcaccaggGuauuucaguuc....................  28     1  Fm2
......................................................................aagAaccaggcuauuucaguuc....................  6      1  Fm2
......................................................................aagcaUcaggcuauuucaguuc....................  6      1  Fm2
......................................................................aagcacUaggcuauuucaguuc....................  4      1  Fm2
......................................................................aagcaccaggUuauuucaguuc....................  21     1  Fm2
......................................................................aagcaccagUcuauuucaguuc....................  4      1  Fm2
......................................................................aagcaccaggcuauuucaguuc....................  23857  0  Fm2
......................................................................aUgcaccaggcuauuucaguuc....................  1      1  Fm2
......................................................................aagUaccaggcuauuucaguuc....................  1      1  Fm2
......................................................................aagcaccaggcuauuucCguuc....................  72     1  Fm2
......................................................................aagcaccaggcuauuucaguCc....................  1      1  Fm2
......................................................................aagcaccaggcuauuucaguuU....................  103    1  Fm2
......................................................................aagcaccaggcuauuucUguuc....................  74     1  Fm2
......................................................................aagcaccaggcuUuuucaguuc....................  6      1  Fm2
......................................................................aagcaccagAcuauuucaguuc....................  3      1  Fm2
......................................................................aagcaccaggcuauuucagAuc....................  1      1  Fm2
......................................................................aagcaNcaggcuauuucaguuc....................  6      1  Fm2
......................................................................aagcaccaggcuauuuAaguuc....................  110    1  Fm2
......................................................................aagcacAaggcuauuucaguuc....................  5      1  Fm2
......................................................................aGgcaccaggcuauuucaguuc....................  4      1  Fm2
......................................................................aagcacGaggcuauuucaguuc....................  2      1  Fm2
......................................................................aNgcaccaggcuauuucaguuc....................  1      1  Fm2
......................................................................Nagcaccaggcuauuucaguuc....................  12     1  Fm2
......................................................................aagcaccaggcuauAucaguuc....................  2      1  Fm2
......................................................................aaCcaccaggcuauuucaguuc....................  3      1  Fm2
......................................................................aagcaccaggcuauuucaguuA....................  25     1  Fm2
......................................................................aaUcaccaggcuauuucaguuc....................  8      1  Fm2
......................................................................aagcaccaggcuauuGcaguuc....................  1      1  Fm2
......................................................................aagcaccCggcuauuucaguuc....................  1      1  Fm2
......................................................................aagcaccaggcuauuucaUuuc....................  5      1  Fm2
......................................................................Gagcaccaggcuauuucaguuc....................  2      1  Fm2
......................................................................aagcaccaggcuaCuucaguuc....................  8      1  Fm2
......................................................................aagcaccaggcuauuucaguucA...................  1347   1  Fm2
......................................................................aagcaccaggcuGuuucaguucu...................  1      1  Fm2
......................................................................aagcaccaggcuauuucaUuucu...................  1      1  Fm2
......................................................................aagcaccaggcuauuuUaguucu...................  2      1  Fm2
......................................................................aagcaccaggcuauuucUguucu...................  5      1  Fm2
......................................................................aagcaAcaggcuauuucaguucu...................  1      1  Fm2
......................................................................aagcaccaggcuauuucaguucG...................  9      1  Fm2
......................................................................aagcaccaggcuauuucaguucC...................  116    1  Fm2
```

Star **Mature**

agccagaacggagcugcugaaccuauccaguggacugggauuccuugugugcuguuugucauaucuuccaaagcaccaggcuauuucaguucucugguugggagaauuccac			
....aagcaccaggcuauuuAaguucu...................	9	1	Fm2
....aagcaccaggGuauuucaguucu...................	5	1	Fm2
....aagcaccaggUuauuucaguucu...................	1	1	Fm2
....aagcaccaggcuauuuGaguucu...................	2	1	Fm2
....aagcaccaUgcuauuucaguucu...................	1	1	Fm2
....aaUcaccaggcuauuucaguucu...................	2	1	Fm2
....aagcaUcaggcuauuucaguucu...................	1	1	Fm2
....aagcaccaggcuauuucGguucu...................	4	1	Fm2
....aagcaccaggcuauuucaguucu...................	2071	0	Fm2
....aagcaccaggcuauuucCguucu...................	6	1	Fm2
....Uagcaccaggcuauuucaguucu...................	2	1	Fm2
....aagcaccaggAuauuucaguucu...................	6	1	Fm2
....aagcaccaggcuaAuucaguucu...................	1	1	Fm2
....aagcaccaggcuauuucaguucuU..................	118	1	Fm2
....aagcaccaggcuauuucaguucuUu.................	22	1	Fm2
....agcaccaggcuauuucaguuc....................	8	0	Fm2
..............................uggacugggaAuccuugugugcu...	1	1	Mm1
..............................uggacugggauuccuugugugcu...	12	0	Mm1
..............................uggacugggauuccuugGgugcu...	1	1	Mm1
..............................uggacugggauuccuugCgugcu...	1	1	Mm1
..............................uggacugggauuccuugugugcuU..	6	1	Mm1
.................................acugggauuccuuguguAcuguuu...	1	1	Mm1
.................................acugggauuccuuguguCcuguuu...	1	1	Mm1
.................................acugggauuccuugugugcuguuu...	15	0	Mm1
.................................acugggauuccuugugugcuguuug..	10	0	Mm1
.................................acugggauuccuugugugcuguuugucauaucuucc...	1	0	Mm1
....ucauaucuuccaaagcaccaggcu..............................	4	0	Mm1
....ucauaucuuccaaagcaUcaggcu..............................	1	1	Mm1
....uccaaagcaccaggcuauuucaguucuc..................	5	0	Mm1
....aaagcaccaggcuauuucaguuc....................	10	0	Mm1
....aagcaccaggcuCuuuca........................	1	1	Mm1
....aagcaccaggcuauuucU........................	6	1	Mm1
....aagcaccaggcuauuuca........................	69	0	Mm1
....aagcaccaggcuauuucaU.......................	1	1	Mm1
....aagcaccaggcuauuucag.......................	32	0	Mm1
....aagcCccaggcuauuucag.......................	1	1	Mm1
....aagcaccaggcuauuuAagu......................	2	1	Mm1
....aagcaccaggcuauuucUgu......................	1	1	Mm1
....aagcaccaggcuauuucagu......................	154	0	Mm1
....aagcaccaggcuauuuGagu......................	2	1	Mm1
....aagcaccaggcuauuuUaguu.....................	3	1	Mm1
....aagcaccaggcuauuucCguu.....................	7	1	Mm1
....aagAaccaggcuauuucaguu.....................	1	1	Mm1
....aagcaccaggUuauuucaguu.....................	2	1	Mm1
....aagcaccaggcuauuucaguG.....................	1	1	Mm1
....Nagcaccaggcuauuucaguu.....................	1	1	Mm1
....aagcaccaggAuauuucaguu.....................	1	1	Mm1
....aagcaccaggcuauuucaguA.....................	7	1	Mm1
....aagcaccaggcuauuucUguu.....................	2	1	Mm1
....aagcaccaggcuauuucaguu.....................	722	0	Mm1
....aagcaccaggcuauuuGaguu.....................	1	1	Mm1
....aagcaccaggcuauuuAaguu.....................	3	1	Mm1
....aagcaccaggcuaAuucaguu.....................	1	1	Mm1
....aagcaccaggcuaCuucaguu.....................	1	1	Mm1
....aagcaccaggGuauuucaguu.....................	1	1	Mm1
....Uagcaccaggcuauuucaguu.....................	2	1	Mm1
....aagcaccaggcuauuuUaguuc....................	20	1	Mm1
....aagcaccaggcuauuucCguuc....................	20	1	Mm1
....aagcaccagCcuauuucaguuc....................	1	1	Mm1
....aagcCccaggcuauuucaguuc....................	1	1	Mm1
....aagcaccagUcuauuucaguuc....................	2	1	Mm1
....aaUcaccaggcuauuucaguuc....................	3	1	Mm1
....aagcaccaggcuaGuucaguuc....................	1	1	Mm1
....aagcaccaggcuauAucaguuc....................	1	1	Mm1
....aaAcaccaggcuauuucaguuc....................	1	1	Mm1
....aagGaccaggcuauuucaguuc....................	2	1	Mm1
....aagcaccaggAuauuucaguuc....................	9	1	Mm1
....aagcaccGggcuauuucaguuc....................	2	1	Mm1
....aagcacUaggcuauuucaguuc....................	2	1	Mm1

```
                                Star                               Mature
agccagaacggagcugcugaaccuauccaguggacugggauuccuugugugcuguuugucauaucuuccaaagcaccaggcuauuucaguucucugguugggagaauuccac
......................................................................aagcaccaggcGauuucaguuc....................   1      1    Mm1
......................................................................Uagcaccaggcuauuucaguuc....................   7      1    Mm1
......................................................................aagcaccaggcuauuucaUuuc....................   2      1    Mm1
......................................................................aagcaccaggUuauuucaguuc....................   5      1    Mm1
......................................................................aaCcaccaggcuauuucaguuc....................   1      1    Mm1
......................................................................aagcaccaggcuauuucaguAc....................   2      1    Mm1
......................................................................aagcGccaggcuauuucaguuc....................   2      1    Mm1
......................................................................aagcaccaggcuauuuGaguuc....................   15     1    Mm1
......................................................................Gagcaccaggcuauuucaguuc....................   1      1    Mm1
......................................................................Nagcaccaggcuauuucaguuc....................   9      1    Mm1
......................................................................aagcaccaggcCauuucaguuc....................   1      1    Mm1
......................................................................aagcaccaggGuauuucaguuc....................   6      1    Mm1
......................................................................aagcaccaggcuauGucaguuc....................   1      1    Mm1
......................................................................aagcaccaggcuauuucaguuc....................   7960   0    Mm1
......................................................................aagcaccaggcuauuuAaguuc....................   29     1    Mm1
......................................................................aagcaNcaggcuauuucaguuc....................   2      1    Mm1
......................................................................aagcaccaggcuauuucaguuU....................   19     1    Mm1
......................................................................aagcaccaggcuauCucaguuc....................   2      1    Mm1
......................................................................aagcaccaggcuaAuucaguuc....................   3      1    Mm1
......................................................................aagcaccaggcuauuucaguuG....................   1      1    Mm1
......................................................................aagcaccaggcuauuCcaguuc....................   1      1    Mm1
......................................................................aagcaccUggcuauuucaguuc....................   1      1    Mm1
......................................................................aagcaccaggcuauuucUguuc....................   27     1    Mm1
......................................................................aGgcaccaggcuauuucaguuc....................   2      1    Mm1
......................................................................aUgcaccaggcuauuucaguuc....................   2      1    Mm1
......................................................................aagcaccaggcuaCuucaguuc....................   1      1    Mm1
......................................................................aagAaccaggcuauuucaguuc....................   2      1    Mm1
......................................................................aagcacAaggcuauuucaguuc....................   1      1    Mm1
......................................................................aagcaccaggcuauuucGguuc....................   19     1    Mm1
......................................................................aagcaccaggcuauuuAaguucu...................   1      1    Mm1
......................................................................aagcaccaggcuauuuUaguucu...................   1      1    Mm1
......................................................................aagcaccaggcuauuuGaguucu...................   2      1    Mm1
......................................................................aagcaccaggcuauuucaguucA...................   455    1    Mm1
......................................................................aagcaccaggcuauuucUguucu...................   1      1    Mm1
......................................................................aagcaccaggcuauuucaguucG...................   10     1    Mm1
......................................................................aagcaccaggcuauuucaguucC...................   34     1    Mm1
......................................................................aagcaccaggUuauuucaguucu...................   1      1    Mm1
......................................................................aagcaccaggAuauuucaguucu...................   1      1    Mm1
......................................................................Nagcaccaggcuauuucaguucu...................   1      1    Mm1
......................................................................Cagcaccaggcuauuucaguucu...................   1      1    Mm1
......................................................................aagcaccaggcuauuucGguucu...................   1      1    Mm1
......................................................................aagcaccaggcuauuucaguucu...................   394    0    Mm1
......................................................................aGgcaccaggcuauuucaguucu...................   1      1    Mm1
......................................................................aagcaccaggcuauuucaguucAc..................   2      1    Mm1
......................................................................aagcaccaggcuauuucaguucuU..................   9      1    Mm1
......................................................................aagcaccaggcuauuucaguucuUu.................   1      1    Mm1
.......................................................................agcaccaggcuauuucagu......................   7      0    Mm1
.......................................................................agcaccaggcuauuucaguuc....................   11     0    Mm1

..............................uggacugggauuccuugugugc............................................................   1      0    Mm2
..............................uggacugggauuccuugugugcG...........................................................   1      1    Mm2
..............................uggacugggauuccuugugugcu...........................................................   4      0    Mm2
.................................acugggauuccuugugugcug..........................................................   3      0    Mm2
.................................acugggauuccuugugugcuguu........................................................   5      0    Mm2
.................................acugggauuccuugugugcuguuu.......................................................   1      0    Mm2
.................................acugggauuccuuguguAcuguuu.......................................................   1      1    Mm2
.................................acugggauuccuugugugcuguuug......................................................   3      0    Mm2
.................................acugggauuccuugugugcuguuugA.....................................................   2      1    Mm2
.................................acugggauuccuugugugcuguuugucau..................................................   1      0    Mm2
.................................acugggauuccuugugugcuguuugucauaucuucca..........................................   1      0    Mm2
...........................................cuugugugcuguuugucauaucuucca..........................................   1      0    Mm2
..........................................................ucauaucuuccaaagcaccaggc...............................   4      0    Mm2
..........................................................ucauaucuuccaaagcaccaggcu..............................   6      0    Mm2
..........................................................ucauaucuuccaaagcaccaggcuauuucaguucu...................   1      0    Mm2
..........................................................ucauaucuuccaaagcaccaggcuauuucGguucu...................   1      1    Mm2
.....................................................................aaagcaccaggcuauuuc.........................   1      0    Mm2
.....................................................................aaagcaccaggcuauuucagu......................   1      0    Mm2
.....................................................................aaagcaccaggcuauuucaguu.....................   2      0    Mm2
.....................................................................Caagcaccaggcuauuucaguuc....................   12     1    Mm2
.....................................................................aaagcaccagAcuauuucaguuc....................   1      1    Mm2
```

Star   Mature

```
agccagaacggagcugcugaaccuauccaguggacugggauuccuugugugcuguuugucauaucuuccaaagcaccaggcuauuucaguucucugguugggagaauuccac
.....................................................................aaagcaccaggcuauuucaguuU....................   2     1   Mm2
.....................................................................aaagcaccaggcuauuucaguuc....................   50    0   Mm2
.....................................................................aaagcaccaggcuauuucaguucu...................   10    0   Mm2
......................................................................aagcaccaggcuauuuca........................   47    0   Mm2
......................................................................aagcaccaggcuauuuUa........................   2     1   Mm2
......................................................................aagcaccaggcuauuucG........................   1     1   Mm2
......................................................................aagcaccaggcuauuucU........................   2     1   Mm2
......................................................................aagcaccaggcuauuuAag.......................   1     1   Mm2
......................................................................aagcaccaggcuauuucaA.......................   4     1   Mm2
......................................................................aagcaccaggcuauuucaU.......................   1     1   Mm2
......................................................................aagcacUaggcuauuucag.......................   1     1   Mm2
......................................................................aagcaccaggcuauuucag.......................   104   0   Mm2
......................................................................aagcaccaggcuauuucCgu......................   4     1   Mm2
......................................................................aagcaccaggcuauuucagA......................   1     1   Mm2
......................................................................aagcaccaggcuauuucagu......................   196   0   Mm2
......................................................................aagcaAcaggcuauuucagu......................   1     1   Mm2
......................................................................aagcaccaggcuauuucGguu.....................   3     1   Mm2
......................................................................aGgcaccaggcuauuucaguu.....................   1     1   Mm2
......................................................................aagcaUcaggcuauuucaguu.....................   2     1   Mm2
......................................................................aagcaccaggcuauuucaguu.....................   1398  0   Mm2
......................................................................aagcaccaggcuauuCcaguu.....................   1     1   Mm2
......................................................................aagcaccaggcuauuucaguG.....................   1     1   Mm2
......................................................................aagcaccaggAuauuucaguu.....................   2     1   Mm2
......................................................................aagcaccaggcuauuuUaguu.....................   3     1   Mm2
......................................................................aagcaccaggcuauuucUguu.....................   8     1   Mm2
......................................................................aagcaccaggcuauCucaguu.....................   1     1   Mm2
......................................................................aaCcaccaggcuauuucaguu.....................   1     1   Mm2
......................................................................aagcaccaggcuauuucaguC.....................   1     1   Mm2
......................................................................aagcacUaggcuauuucaguu.....................   1     1   Mm2
......................................................................aagcaccaggcuauuucagCu.....................   1     1   Mm2
......................................................................aagcaccaggGuauuucaguu.....................   1     1   Mm2
......................................................................aagcaccaggcuauuuGaguu.....................   3     1   Mm2
......................................................................aagcaccaggcuauuuAaguu.....................   6     1   Mm2
......................................................................aagcaccaggcuauuucCguu.....................   5     1   Mm2
......................................................................aagcUccaggcuauuucaguu.....................   1     1   Mm2
......................................................................aagcaccaggcuauuucaguA.....................   10    1   Mm2
......................................................................aagUaccaggcuauuucaguu.....................   1     1   Mm2
......................................................................aagcaccaggcuaAuucaguuc....................   14    1   Mm2
......................................................................aGgcaccaggcuauuucaguuc....................   7     1   Mm2
......................................................................aagcaccaggcuauuucGguuc....................   129   1   Mm2
......................................................................aagcaccaggcuCuuucaguuc....................   2     1   Mm2
......................................................................aagcacNaggcuauuucaguuc....................   8     1   Mm2
......................................................................aagcaccaggcuaGuucaguuc....................   4     1   Mm2
......................................................................aagcaccaggGuauuucaguuc....................   38    1   Mm2
......................................................................Uagcaccaggcuauuucaguuc....................   15    1   Mm2
......................................................................aagcaccaggUuauuucaguuc....................   30    1   Mm2
......................................................................aagcaccaggcuauuucaguuG....................   1     1   Mm2
......................................................................aagcaGcaggcuauuucaguuc....................   2     1   Mm2
......................................................................aagcaccUggcuauuucaguuc....................   14    1   Mm2
......................................................................aagcaccaggcuGuuucaguuc....................   5     1   Mm2
......................................................................aagcaccaggcuauuucaguuU....................   203   1   Mm2
......................................................................aagcUccaggcuauuucaguuc....................   2     1   Mm2
......................................................................aagcacGaggcuauuucaguuc....................   4     1   Mm2
......................................................................aagUaccaggcuauuucaguuc....................   4     1   Mm2
......................................................................aagcaccaggcuauuucagGuc....................   2     1   Mm2
......................................................................aagcaAcaggcuauuucaguuc....................   2     1   Mm2
......................................................................aagcacAaggcuauuucaguuc....................   4     1   Mm2
......................................................................aagcaccagCcuauuucaguuc....................   2     1   Mm2
......................................................................aagcCccaggcuauuucaguuc....................   2     1   Mm2
......................................................................aUgcaccaggcuauuucaguuc....................   7     1   Mm2
......................................................................Gagcaccaggcuauuucaguuc....................   9     1   Mm2
......................................................................aagcaccaggcuauuuGaguuc....................   60    1   Mm2
......................................................................aagcaccagAcuauuucaguuc....................   4     1   Mm2
......................................................................Cagcaccaggcuauuucaguuc....................   3     1   Mm2
......................................................................aaCcaccaggcuauuucaguuc....................   3     1   Mm2
......................................................................aagcaccaggcuUuuucaguuc....................   8     1   Mm2
......................................................................aagcGccaggcuauuucaguuc....................   3     1   Mm2
......................................................................aagcaNcaggcuauuucaguuc....................   11    1   Mm2
......................................................................aagcacUaggcuauuucaguuc....................   12    1   Mm2
......................................................................aagcaccGggcuauuucaguuc....................   8     1   Mm2
```

```
                                                  Star                                Mature
agccagaacggagcugcugaaccuauccaguggacugggauuccuugugugcuguuugucauaucuuccaaagcaccaggcuauuucaguucucugguugggagaauuccac
......................................................................aCgcaccaggcuauuucaguuc....................    2      1    Mm2
......................................................................aagcaccaggcuauuucaguuc....................    43690  0    Mm2
......................................................................aaAcaccaggcuauuucaguuc....................    16     1    Mm2
......................................................................aagcaccaggcuauGucaguuc....................    1      1    Mm2
......................................................................Nagcaccaggcuauuucaguuc....................    22     1    Mm2
......................................................................aagcaccaggcuauuCcaguuc....................    9      1    Mm2
......................................................................aagcaccaggcuauuucaguCc....................    8      1    Mm2
......................................................................aagcaccaggcGauuucaguuc....................    3      1    Mm2
......................................................................aagcaccaggcuauCucaguuc....................    3      1    Mm2
......................................................................aagcaccaggcuaCuucaguuc....................    7      1    Mm2
......................................................................aagcaccaCgcuauuucaguuc....................    1      1    Mm2
......................................................................aagcaccaggcuauuucagAuc....................    3      1    Mm2
......................................................................aagcaccaggcCauuucaguuc....................    4      1    Mm2
......................................................................aaUcaccaggcuauuucaguuc....................    17     1    Mm2
......................................................................aagcaUcaggcuauuucaguuc....................    12     1    Mm2
......................................................................aagcaccaggcuauuucaAuuc....................    4      1    Mm2
......................................................................aagcaccaggcuauuucagCuc....................    6      1    Mm2
......................................................................aagcaccaggcuauuucUguuc....................    138    1    Mm2
......................................................................aagcaccaggAuauuucaguuc....................    94     1    Mm2
......................................................................aagAaccaggcuauuucaguuc....................    5      1    Mm2
......................................................................aagcaccaggcAauuucaguuc....................    2      1    Mm2
......................................................................aagcaccaUgcuauuucaguuc....................    8      1    Mm2
......................................................................aagcaccaggcuauuAcaguuc....................    7      1    Mm2
......................................................................aagcaccaggcuauAucaguuc....................    2      1    Mm2
......................................................................aagcaccaggcuauuucaguGc....................    2      1    Mm2
......................................................................aagcaccaggcuauuucaguuA....................    14     1    Mm2
......................................................................aagcaccaggcuauuucaUuuc....................    6      1    Mm2
......................................................................aagcaccaggcuauuucCguuc....................    133    1    Mm2
......................................................................aagcaccaggcuauuUaguuc....................    114    1    Mm2
......................................................................aagcaccagUcuauuucaguuc....................    3      1    Mm2
......................................................................aagcaccaAgcuauuucaguuc....................    4      1    Mm2
......................................................................aagcaccaggcuauuucaguAc....................    4      1    Mm2
......................................................................aagcaccaggcuauuUAaguuc....................    191    1    Mm2
......................................................................aagcaccCggcuauuucaguuc....................    4      1    Mm2
......................................................................aagcaccaggcuauuucaguucu...................    2107   0    Mm2
......................................................................aagcaccaggcuauCucaguucu...................    1      1    Mm2
......................................................................aagcaccaggGuauuucaguucu...................    2      1    Mm2
......................................................................aagcaccaggcuauuucUguucu...................    8      1    Mm2
......................................................................aagcaccaggcuauuucCguucu...................    7      1    Mm2
......................................................................aagcaccaggcuauuUaguucu...................    11     1    Mm2
......................................................................aagcaccaggcuauuucaguucC...................    93     1    Mm2
......................................................................aagcaccaggcuauuucaguuAu...................    1      1    Mm2
......................................................................aagcaccaggcuauuucaguucG...................    15     1    Mm2
......................................................................aagcaccaggcuauuCcaguucu...................    1      1    Mm2
......................................................................aagcaccGggcuauuucaguucu...................    1      1    Mm2
......................................................................aaCcaccaggcuauuucaguucu...................    1      1    Mm2
......................................................................aagcaccaggcuauuucaguucA...................    1549   1    Mm2
......................................................................aagcaccaggUuauuucaguucu...................    5      1    Mm2
......................................................................aagcaccaUgcuauuucaguucu...................    1      1    Mm2
......................................................................aagcaNcaggcuauuucaguucu...................    1      1    Mm2
......................................................................aagcaccaggcuauuuAaguucu...................    5      1    Mm2
......................................................................aagcaccaggcuaCuucaguucu...................    1      1    Mm2
......................................................................aGgcaccaggcuauuucaguucu...................    1      1    Mm2
......................................................................aagcaccaggcuauuucGguucu...................    7      1    Mm2
......................................................................aagcaccagUcuauuucaguucu...................    1      1    Mm2
......................................................................aagcaccaggAuauuucaguucu...................    3      1    Mm2
......................................................................aagcaccaggcuauuucaguuUu...................    10     1    Mm2
......................................................................aagcGccaggcuauuucaguucu...................    1      1    Mm2
......................................................................aagcaccaggcuauuucaguucAc..................    1      1    Mm2
......................................................................aagcaccaggcuauuucaguucuU..................    76     1    Mm2
......................................................................aagcaccaggcuauuucaguucuA..................    3      1    Mm2
......................................................................aagcaccaggcuauuucaguucuUu.................    16     1    Mm2
.......................................................................agcaccaggcuauuucagu......................    1      0    Mm2
.......................................................................agcaccaggcuauuucaguuc....................    16     0    Mm2
.......................................................................agcaccaggcuauuucaguucu...................    2      0    Mm2
```
